# *BCoR-L1 *variation and breast cancer

**DOI:** 10.1186/bcr1759

**Published:** 2007-08-16

**Authors:** Felicity Lose, Jeremy Arnold, David B Young, Carolyn J Brown, Graham J Mann, Gulietta M Pupo, The Kathleen Cuningham Foundation Consortium for Research into Familial Breast Cancer, Kum Kum Khanna, Georgia Chenevix-Trench, Amanda B Spurdle

**Affiliations:** 1 Cancer and Cell Biology Division, Queensland Institute of Medical Research, 300 Herston Road, Brisbane, Queensland, Australia, 4006; 2 School of Medicine, Central Clinical Division, University of Queensland, Royal Brisbane Hospital, Corner Butterfield Street and Bowen Bridge Road, Brisbane, Queensland, Australia, 4029; 3 Department of Medical Genetics, Molecular Epigenetics Group, University of British Columbia, 2329 West Mall, Vancouver, BC, Canada, V6T 1Z4; 4 Westmead Institute for Cancer Research, University of Sydney at Westmead Millennium Institute, Westmead Hospital, Darcy Road, Westmead, New South Wales, Australia, 2145

## Abstract

**Introduction:**

BRCA1 is involved in numerous essential processes in the cell, and the effects of BRCA1 dysfunction in breast cancer carcinogenesis are well described. Many of the breast cancer susceptibility genes such as *BRCA2*, *p53*, *ATM*, *CHEK2*, and *BRIP1 *encode proteins that interact with BRCA1. BCL6 corepressor-like 1 (BCoR-L1) is a newly described BRCA1-interacting protein that displays high homology to several proteins known to be involved in the fundamental processes of DNA damage repair and transcription regulation. BCoR-L1 has been shown to play a role in transcription corepression, and expression of the X-linked *BCoR-L1 *gene has been reported to be dysregulated in breast cancer subjects. *BCoR-L1 *is located on the X chromosome and is subject to X inactivation.

**Methods:**

We performed mutation analysis of 38 *BRCA1/2 *mutation-negative breast cancer families with male breast cancer, prostate cancer, and/or haplotype sharing around *BCoR-L1 *to determine whether there is a role for *BCoR-L1 *as a high-risk breast cancer predisposition gene. In addition, we conducted quantitative real-time PCR (qRT-PCR) on lymphoblastoid cell lines (LCLs) from the index cases from these families and a number of cancer cell lines to assess the role of *BCoR-L1 *dysregulation in cancer and cancer families.

**Results:**

Very little variation was detected in the coding region, and qRT-PCR analysis revealed that *BCoR-L1 *expression is highly variable in cancer-free subjects, high-risk breast cancer patients, and cancer cell lines. We also report the investigation of a new expression control, *DIDO1 *(death inducer-obliterator 1), that is superior to *GAPDH *(glyceraldehyde-3-phosphate dehydrogenase) and *UBC *(ubiquitin C) for analysis of expression in LCLs.

**Conclusion:**

Our results suggest that *BCoR-L1 *expression does not play a large role in predisposition to familial breast cancer.

## Introduction

Less than 40% of familial breast cancer can be attributed to mutations in the high-risk genes *BRCA1 *and *BRCA2 *despite their high penetrance [1,2]. Syndromes displaying a predisposition for breast cancer such as Li-Fraumeni syndrome (resulting from *p53 *gene mutations) [3], ataxia telangiectasia (ataxia telangiectasia-mutated, or *ATM*, gene) [4], and Cowden syndrome (phosphatase and tensin homologue, or *PTEN*, gene) [5] are estimated to account for no more than 10% of familial breast cancer collectively, and additional moderate-risk genes such as *CHEK2 *[6] and the recently reported *BRIP1 *(also called *BACH1*) [7] and *PALB2 *[8,9] account for an even smaller percentage. This leaves a large proportion of the genetic basis of familial breast cancer unexplained.

Interestingly, BRCA2, p53, ATM, CHEK2, and BRIP1 all interact with the multifunctional tumour suppressor, BRCA1. BRCA1-interacting proteins are logical breast cancer candidates for two reasons. First, they are likely to be involved in some of the important roles of BRCA1 such as genome maintenance, transcription regulation, and cell cycle control and, if mutated, may result in the same highly penetrant and damaging effect as a *BRCA1 *mutation. Second, mutations in these interacting genes may prevent BRCA1 from performing vital functions, resulting in the same acute effect as a *BRCA1 *mutation itself. Recently, Pagan and colleagues [10] described the characterisation and functional analysis of a novel BRCA1-interacting protein, BCL6 corepressor-like 1 (BCoR-L1), that displays homology to several proteins involved in pathways such as DNA damage repair (BARD1 and *Drosophila *recombination repair protein 1) and transcription regulation (BCoR). Functional analysis thus far has revealed a role for BCoR-L1 in transcriptional corepression [10], placing it in a large group of proteins involved in the regulation of proliferation and apoptosis [11]. It is well established that uncontrolled overexpression of oncogenes and repression or mutation of tumour suppressors contribute to tumourigenesis by disturbing these vitally important and tightly controlled cellular processes [12].

Evidence that BCoR-L1 operates primarily as a transcription corepressor includes its ability to dramatically reduce reporter gene expression through an interaction with the CtBP (carboxyl-terminal binding protein) corepressor via a PLDLS motif and the fact that it associates with a number of class II histone deacetylases (HDACs), factors also involved in transcription repression [10]. Similarly, BRCA1 interacts with a number of proteins involved in chromatin remodelling and transcription control [13]. Like BRCA1, BCoR-L1 is also involved in maintaining genomic stability after DNA damage.

In addition to its interaction with BRCA1, there is indirect evidence to suggest that BCoR-L1 may behave as a tumour suppressor. The BCoR-L1 protein appears to be expressed ubiquitously at low levels (including breast tissue), with high levels in reproductive tissues such as prostate and testes [10]. However, *BCoR-L1 *expression was found to be decreased in a variety of breast cancer subjects, including *BRCA1/2 *mutation carriers and 'sporadic' breast cancer subjects [14]. In addition, the *BCoR-L1 *gene is located at Xq26.1, a region reported to exhibit loss of heterozygosity (LOH) in many tumour types, including those of the breast [15-17]. *BCoR-L1 *is subject to complete X inactivation [18], and interestingly, an increased frequency of skewed X inactivation has been reported in both ovarian [19] and early-onset *BRCA1/2 *mutation-negative (*BRCAX*) breast [20,21] cancer populations. It has been proposed that skewed X inactivation could provide a novel mechanism for nonrandom expression of a mutant tumour suppressor gene and thereby contribute to tumourigenesis.

We sought to determine whether there is a role for *BCoR-L1 *as a high-risk breast cancer predisposition gene by screening the coding region of the gene in 38 *BRCAX *breast cancer families by means of the highly sensitive mutation detection technique, denaturing high-performance liquid chromatography (DHPLC). The majority of families were chosen specifically for the presence of male breast cancer and/or early-onset prostate cancer, in combination with being a high-risk female breast cancer family, for two reasons. We hypothesised that an X-linked gene may be involved in these male cancer because males carry only one copy of the X chromosome and because *BCoR-L1 *has been found to be expressed highly in the prostate [10]. A small number of breast cancer families in which the affected individuals showed haplotype sharing around the *BCoR-L1 *locus were also included in this study. In addition, we conducted quantitative real-time PCR (qRT-PCR) on lymphoblastoid cell lines (LCLs) from members of these families and a number of cancer cell lines to assess the role of *BCoR-L1 *expression in cancer and cancer families. We assessed *BCoR-L1 *expression in LCLs from breast cancer cases to determine whether *BCoR-L1 *expression was altered in familial breast cancer cases and, if so, whether this was associated with the presence of genetic variation. Analysis of X inactivation status of *BCoR-L1 *was also undertaken in order to assess the likely mode of inheritance of *BCoR-L1 *as a candidate tumour suppressor gene.

## Materials and methods

### X inactivation status analysis

X inactivation status was assessed by comparison of *BCoR-L1 *mRNA expression with human-specific primers (forward: CATATGATGTGACGGAATCTC; reverse: CCCTGGACTTTGTTGGGCA) in mouse-human or hamster-human hybrid cell lines containing a human active X chromosome (AHA-11aB1, A23-1aC1I5, t60-12, GM06318D, CHO-01416-M) or a human inactive X chromosome (LT23-1E2, t48-1a-1Daz4a, t11-4Aaz5, t75-2maz34-1a, t86-B1maz1b-3a, X8-6T2S1, CHO-01416-07). Comparison of expression levels between the two groups of cell lines (containing an inactive versus inactive human X chromosome) was used to establish whether *BCoR-L1 *is subject to X inactivation.

### Subjects

Multiple-case breast cancer families were ascertained through the Kathleen Cuningham Foundation Consortium for Research into Familial Breast Cancer (kConFab) [22]. Inclusion criteria for all families in this study required that the family be classified as category 3 (high-risk) according to the National Breast Cancer Centre guidelines [23] and that the family not possess any known mutations in the *BRCA1 *or *BRCA2 *genes (*BRCAX*) at the time of initiation of the study. Ethics approvals were obtained from the ethics committees of the Peter MacCallum Cancer Institute (East Melbourne, Victoria, Australia) and the Queensland Institute of Medical Research (Brisbane, Queensland, Australia), and all subjects gave written informed consent. Male breast cancer families selected for study (*n *= 21) all contained one or more male breast cancer cases occurring on the same side of the family as at least three female breast cancer cases. A total of 11 male breast cancer cases and 16 female index cases (the youngest breast cancer case in the family from which biospecimens were available) were screened from these families. Prostate/female breast cancer families (*n *= 12) were chosen to contain at least one 'early-onset' prostate cancer case (cancer diagnosed at not more than 60 years of age) on the same side of the family as three or more female breast cancer cases. A total of 2 prostate cancer cases and 12 index female breast cancer cases were analysed from these families. Pedigrees were examined to ensure the absence of male-to-male transmission of the disease in these families because this would imply involvement of an autosomal gene. Additionally, families with female breast cancer only (*n *= 7) were selected for analysis because they demonstrated haplotype sharing in the same chromosomal region as *BCoR-L1 *(DXS1001, DXS1047, and DXS1227; LOD [logarithm of the odds] score greater than 0.5; data not shown) and are referred to as *BCoR-L1 *haplotype sharing families. Two *BCoR-L1 *variants were found in one family and were then genotyped in all family members from which biospecimens were available (*n *= 12). Mutation screening in the kConFab cohort during the course of the study subsequently identified *BRCA2 *mutation carriers in two male breast cancer families. Control subjects (46 females and 55 males) without cancer or a family history of cancer included subjects ascertained via the Queensland Blood Bank and a group of geriatric controls (average age of 80 years).

### Screening for *BCoR-L1 *variation

BCoR-L1 is expressed in two isoforms. The most common isoform is 1,711 amino acids in size and lacks exon 9, but the full-length protein (1,785 amino acids long) is derived from the alternative transcript. This study screened the entire coding region of the *BCoR-L1 *gene, including exon 9. Primers encompassing the 13 coding exons of *BCoR-L1 *(and surrounding intronic regions; GenBank: exons 2 to 8: Z82208; exons 9 to 14: AL136450) were designed using Primer3 [24] (Table 1). Exon 4 was too large to be amplified at an optimal size for DHPLC analysis and was therefore analysed with 10 overlapping polymerase chain reaction (PCR) fragments. 'Standard' PCR reactions were carried out in a 20-μl mixture containing 15 ng of genomic DNA, and a final concentration of 20 pmol of each primer, 200 μM each of dATP, dCTP, dGTP, and dTTP (Promega Corporation, Madison, WI, USA), 1.5 mM MgCl_2_, 1× PCR buffer, and 1 U Ampli*Taq *Gold polymerase (PE Applied Biosystems, Foster City, CA, USA). Any variation to the reaction is detailed in Table 1, along with a description of 'touchdown' PCR amplification conditions. All products (and H_2_O controls) were visualised on a 1.5% agarose gel. Male samples were mixed with sequence-confirmed wild-type female PCR product (2:1) to encourage heteroduplexes to form for successful DHPLC analysis. Samples were denatured by heating to 95°C for 5 minutes and cooling to 60°C over a period of 30 minutes and then analysed on a Varian Helix DHPLC system (Varian, Inc., Palo Alto, CA, USA) at the recommended melt temperature(s) as determined by the Stanford DHPLC Melt program [25] (Table 1). Analysis of results was carried out using Star Workstation Reviewer software (version 5, Varian) and any aberrant or shifted profiles were reamplified for confirmation of the aberrant profile by repeat DHPLC before being sequenced using the Big-Dye (version 3.1) sequencing chemistry and PE Applied Biosystems 377 sequencer.

**Table 1:** *BCOR-L1 *polymerase chain reaction conditions

Exon	PCR fragment	Forward primer	Reverse primer	PCR conditions	Annealing temperature^a ^(°C)	Size (bp)	DHPLC temperature (°C)
2	2	GGCTGGCTGCTTTAACATTC	CTCCCCAGGCCCTATTGTAT	2 U *Taq*, 40 pmol primers, 0.5 M betaine	54	425	62
3	3	AGGTGGTGTTGGCTCAAATC	CAACTCGACCAACCAGGTCT	40 pmol primers	54	404	62
4	4a	TGTGCATGCTATCCTGTCGT	GCTGGCAGAGGACTGAAGTT	40 pmol primers	54	450	62
4	4b	GAACTGGAGTCCCTGTGGAG	GAGGGTGGGGGTAGAAGGT	2 U *Taq*, 1 mM MgCl_2_, 1 M betaine	54	578	63
4	4c	GTCCCCACTCCGGTTCTG	CAGGGAGCGTAAGAGTGGAG	Standard	54	442	63
4	4d	TGGTATATATCCCGCCTCCA	GTCCCTTCTGTTTGCTGCTC	40 pmol primers	54	436	57, 62
4	4e	CTTCCAACTCCACAGCCTCT	AATGGTGCTGATCAGTGCAG	2 mM MgCl_2_, 0.5 M betaine	58	459	62
4	4f	CTCGCCCTTTGTCATCTTTC	GCTGGTAGGTTTCCCATTGA	2 mM MgCl_2_	54	424	62
4	4g	GACAGCCAAGCACAGTGAAA	GCTGAGGGTCAAGAGGACAG	Standard	54	452	62
4	4h	CTCCTTCGTTCCAGAGCAGG	CCAGGACCAGCTCATGGGAC	Standard	59	314	61
4	4i	AGAGAGCCACCTCTGCTCTG	ACCCCTACGCTTTCCTGTTT	Standard	54	435	62
4	4j	AAGGTGGATGGTGATGTGGT	GAGGGGACAGCAGGTCATTA	Standard	54	457	62
5	5	GCAGCTCATGCCTCTAGGTC	ATCCTTGCTCGCTCACCTTA	Standard	54	446	62
6	6	GCAAAAGCGACCAAACTCTC	AATTCCCAACTCGACACCTG	2 U *Taq*	56	423	60
7	7	TCCTCTGTACATCCCATCCAC	GTAGAGATGCCCGAGGGTTC	2 mM MgCl_2_	63	483	62
8	8	AGGCGTTGCTTTTCTGTGTT	CGCCACACACACCTTCTACA	2 mM MgCl_2_	57	332	60
9	9	ATGACCCTGGTGGATGGATA	GGTTCAAGCACCAGAAGAGC	Standard	62	378	61
10	10	TGGGCAACAGAGTGAGACTG	GCAGGCAAGGTCTTTTGAGT	Standard	54	488	62
11	11	CAGGTGGTTCCCTTGTCCTA	GAGCTGTTCAAGGTGGAAGG	Standard	54	399	61
12	12	CTTCTCCCAATTCCCTTAGCC	AAAGCCAGGGAGAAGAAAGG	0.5 M betaine	54	454	60
13	13	CCCCTATATGCTCCCCTTACA	TTGCCAGGTCTTCACTTCCT	Standard	54	273	60
14	14	TTCCTCCAGCCTCCTTCAAT	CCCGGGACCTCTTGTCCT	40 pmol primers	54	595	62

^a^'Touchdown' polymerase chain reaction (PCR) amplification conditions were as follows: denaturation at 94°C for 10 minutes, followed by two cycles of 94°C for 30 seconds, 30 seconds at the fragment annealing temperature (T_A_) + 6°C, and 72°C for 30 seconds. The conditions remained the same for the rest of the PCR except for the T_A_, which consisted of two cycles at (T_A _+ 4°C), then two cycles at (T_A _+ 2°C), and (finally) 35 cycles at the T_A_. A final extension step was conducted at 72°C for 7 minutes. bp, base pairs; DHPLC, denaturing high-performance liquid chromatography.

### Loss of heterozygosity analysis

LOH analysis was carried out on tumour blocks from the *BCoR-L1 *haplotype sharing family carrying the exon 4 c.516T>C variant, because genotyping analysis revealed that the variant segregated with breast cancer. Macrodissected tumour and adjacent cancer-uninvolved tissue DNA was extracted from tumour blocks by means of a modified version of the method of Levi and colleagues [26], and 2 μl of each DNA (plus 20 ng of lymphocyte-derived germline DNA from the same subject) was then added to separate 20-μl PCR reactions, as detailed above. Primers used were (forward) TCAACACCCAAATGAGCAAA and (reverse) GAACAGAGTGGGGCACAGAG to give a product of 242 base pairs. 'Touchdown' PCR was used with an annealing temperature of 50°C. PCR products were then purified (Qiagen Inc., Valencia, CA, USA) and sequenced. LOH was evaluated by scoring the absence of the allele in the sequencing trace of the tumour, compared to matching germline DNA.

### *BCoR-L1 *expression analysis

**Figure 1:**
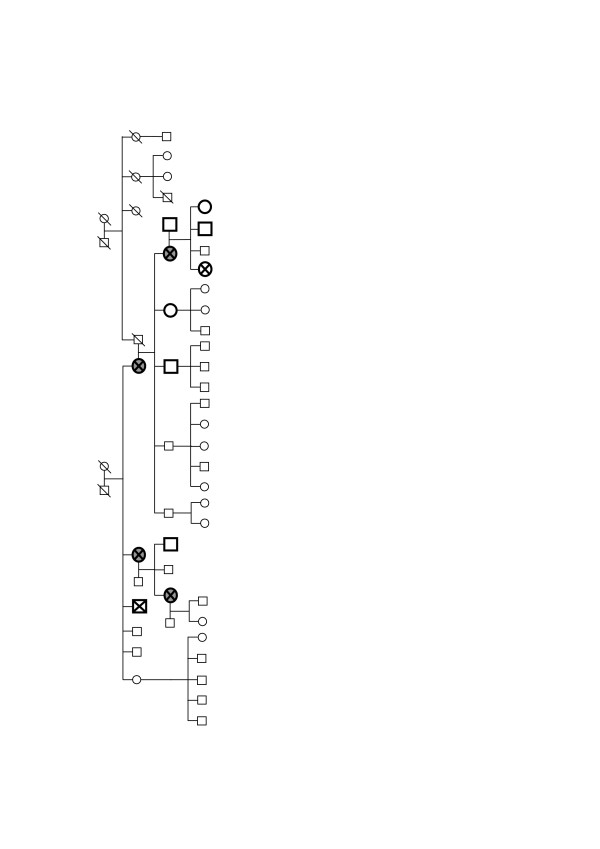
***BCoR-L1 *expression in cancer and normal cell lines**. **(a) ***BCoR-L1 *expression in cancer and normal cell lines. **(b) **Mean and standard deviation of *BCoR-L1 *expression in cancer and normal cell lines. Normal cell lines: ovarian – OSE 64/96, HOSE 17.1; breast – SVCT, Bre80hTERT; prostate – RWPE1. *BCoR-L1*, BCL6 corepressor-like 1; *GAPDH*, glyceraldehyde-3-phosphate dehydrogenase.

All LCLs and normal and cancer cell lines were grown in RPMI 1640 media with 10% fetal calf serum and 1% penicillin/streptomycin. Cell lines used are detailed in Figure 1a. RNA was extracted from LCLs and cell lines using TriReagent (Sigma-Aldrich, St. Louis, MO, USA) according to the manufacturer's instructions, and 1 μg of each sample was placed in a reverse transcription-PCR reaction using Superscript III RNAse H^- ^Reverse Transcriptase as directed by the manufacturer (Invitrogen Corporation, Carlsbad, CA, USA). qRT-PCR was then carried out in a 15 μl reaction containing a final volume of 20 ng of cDNA, 20 pmol of each primer, and 7.5 μl of Platinum SYBR Green qPCR Supermix UDG (Invitrogen Corporation). Primer sequences used are detailed in Table 2. qRT-PCR conditions were 50°C for 2 minutes and 95°C for 2 minutes, 40 cycles of 95°C for 20 seconds, 60°C for 15 seconds, and 72°C for 20 seconds (acquiring) on a Rotor-Gene RG-3000 Real-Time PCR machine (Corbett Research Australia, Mortlake, New South Wales, Australia). All samples were run in duplicate and were repeated if profiles did not replicate according to Rotor-Gene analysis software (version 5). Accordingly, the maximum standard deviation allowed for a pair of duplicates ('Rep. Ct Std. Dev' in analysis software) was low (≤0.2). Expression levels were presented as the mean of two duplicates, normalised to expression of either *GAPDH *(all samples) or *DIDO1 *(death inducer-obliterator 1) (LCLs only). Expression levels of different groups were compared using the Student *t *test (two-tailed).

**Table 2:** Quantitative real-time polymerase chain reaction primer sequences

Gene	GenBank ID	Forward primer	Reverse primer
*BCoR-L1*	AL136450	GACCGACATCCTGAACATCC	ATAGGACAGCAGGAGCCAGA
*GAPDH*	NT_009759	CTGCACCACCAACTGCTTAG	GTCTTCTGGGTGGCAGTGAT
*UBC*	NM_021009	CTTGTTTGTGGATCGCTGTG	GTGTCACTGGGCTCAACCTC
*DIDO1*	NT_011333	GCCTGAATGTGAGGGTTACG	ACAATCGCCATGAAACCATT

*BCoR-L1*, BCL6 corepressor-like 1; *DIDO1*, death inducer-obliterator 1; *GAPDH*, glyceraldehyde-3-phosphate dehydrogenase; *UBC*, ubiquitin C.

### Assessment of expression controls for lymphoblastoid cell lines

RT-PCR expression controls are typically chosen for their stability of expression not only during various phases of the cell cycle, but also between different tissue types. However, it is well known that these characteristics are very difficult to obtain. It has been reported that widely accepted expression controls such as *β-actin *and *GAPDH *show unacceptable variation in expression in a large number of tissues and are therefore not ideal controls [27]. We sought to find a suitable expression control for analysis of expression in LCLs and compared this with two widely used expression controls, *GAPDH *and ubiquitin C (*UBC*). Cheung and colleagues [28] used microarray analysis to establish the variability of expression of 5,184 genes in LCLs taken from random individuals. We evaluated the 100 least variably expressed genes for suitability as an LCL expression control for our project via a PubMed [29] search of the literature to identify the genes that (a) had not been reported to be associated with cancer of any kind and (b) were not in a region of LOH or linkage to any cancer. *DIDO1 *was chosen according to these criteria.

## Results and Discussion

*BCoR-L1 *was selected as an interesting breast cancer candidate gene for a number of reasons. BCoR-L1 interacts with the important breast cancer susceptibility gene product, BRCA1, and there is the possibility that BCoR-L1 is involved, with BRCA1, in critical DNA repair and cell growth pathways. Indeed, mutations in repressor proteins have been implicated in several diseases, including cancer [30]. For example, functional mutations in the transcriptional repressor *Rb *gene result in dysregulation of cell cycle control [31]. In addition, the *BCoR-L1 *gene is located at Xq26.1, a region that exhibits LOH in cancers, including breast cancer, and there is some evidence that *BCoR-L1 *mRNA expression is deregulated in breast cancer. Skewed X inactivation has been reported in breast and ovarian cancer subjects, indicating the possible presence of an X-linked cancer predisposition gene.

### X inactivation status analysis

Analysis of the hybrid cell lines showed that *BCoR-L1 *was not expressed in 4/5 hybrid cell lines containing an inactive X chromosome and was expressed at very low levels in the fifth cell line t48-1a-1Daz4a, using hybrids containing an active X chromosome as reference. These results indicate that *BCoR-L1 *is subject to X chromosome inactivation in humans, which is in agreement with the study of Carrel and Willard [18], who showed that the proximal *UTP14A *gene was expressed in 3/9 hybrids whereas *BCOR-L1 *(FLJ11362) was silent in all 9. As some of the hybrids in this study overlap, *BCOR-L1 *is silenced in 11/11 hybrids studied to date. The fact that *BCoR-L1 *is subject to X inactivation suggests that only a single mutational event in the *BCoR-L1 *gene would be required to initiate tumourigenesis.

### Variation in the *BCoR-L1 *gene

**Figure 2:**
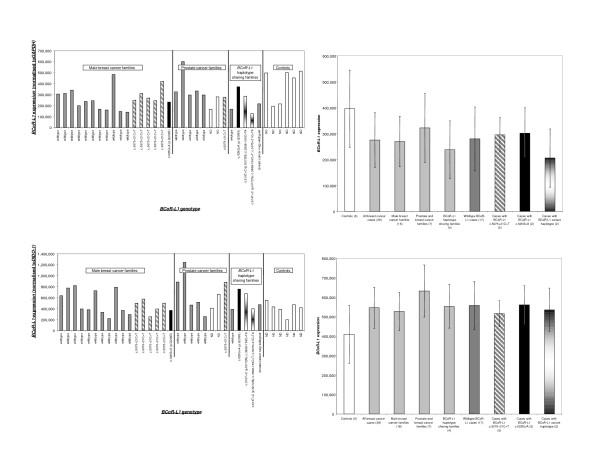
***BCoR-L1 *haplotype sharing family pedigree detailing carriers of the c.516T>C and c.3608-156C>T variants**.  = breast cancer-positive; c.516T>C and c.3608-156C>T-positive.  = breast cancer-negative; c.516T>C and c.3608-156C>T-positive. □ = breast cancer-negative; c.516T>C and c.3608-156C>T-negative. Circle = female, square = male; subjects marked by small shapes were not available for genotyping. *BCoR-L1*, BCL6 corepressor-like 1.

DHPLC analysis of the coding region of *BCoR-L1 *in 48 members of 38 high-risk *BRCAX *breast cancer families revealed only four different sequence variations (Table 3). A nucleotide variation in exon 4 (c.516T>C; p.N172N) was found in one breast cancer family known to share a haplotype at the *BCoR-L1 *locus. This variant was carried primarily by breast cancer cases from this family (Figure 2). However, this c.516T>C nucleotide substitution is not likely to be functional because it does not result in an amino acid change and is not predicted by *in silico *modelling to have any effect on mRNA structure (MFOLD [32]) and the wild-type T allele is not conserved in either mouse or rat (PipMaker [33]). Investigation of exonic splice enhancer (ESE) sites by means of ESEfinder (version 2.0) [34] revealed that the variant allele produces an SC35 site (of value 2.455), but this score is very close to the SC35 threshold of 2.383. Similar analysis using Rescue ESE [35] revealed no change. Additionally, analysis of codon usage of the AAT codon (wild-type) in the human genome versus the AAC codon (variant) showed that the two codons are used at similar frequencies (16.8 versus 19.1/1,000 codons) [36]. Finally, LOH analysis of the c.516T>C variant gave no evidence of the change being involved in tumourigenesis. LOH analysis of tumour blocks and germline DNA from all four breast cancer cases in this family revealed either loss of the variant allele or no LOH (data not shown), indicating that this variant is unlikely to be pathogenic.

**Table 3:** Variation detected in the *BCoR-L1 *gene

Gene region	Polymerase chain reaction fragment	Nucleotide change	Amino acid change	Number of total heterozygous^a ^cases		Male breast cancer families		Prostate cancer families		*BCoR-L1 *haplotype sharing families		Number of heterozygous controls		Previously reported^b^
Exon 4	4a	c.516T>C	p.N172N	2/48	4.2%	-		-		2/9	22.2%	0/101	0%	No
Exon 4	4b	c.625G>A	p.G209S	6/48	12.5%	2/29^c^	6.9%	3/15	20.0%	2/9	22.2%	18/73	24.6%	Yes
Intron 5	6	c.3608-156C>T	-	2/48	4.2%	-		-		2/9	22.2%	0/99	0%	No
Intron 13	13	c.5075+21C>T	-	10/48	20.8%	5/29^c^	17.2%	3/15	20.0%	2/9	22.2%	26/102	25.5%	Yes

Three samples were included in both the male breast cancer family and prostate cancer family categories because they fitted both criteria. Two samples were similarly included in both male breast cancer family and *BCoR-L1 *haplotype sharing family categories. ^a^Males carrying a variant are homozygous. ^b^Reported in dbSNP or SNPper databases. ^c^One of the variant-carrying subjects possesses a mutation in *BRCA2*. *BCoR-L1*, BCL6 corepressor-like 1.

**Figure 3:**
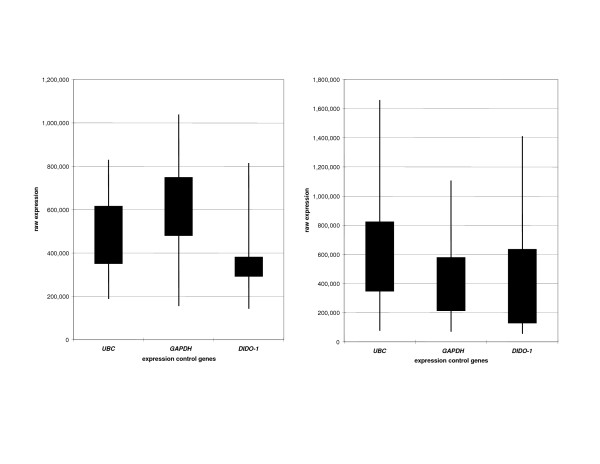
***BCoR-L1 *expression in lymphoblastoid cell lines (LCLs) from breast cancer families**. **(a) ***BCoR-L1 *expression in LCLs from breast cancer families (normalised to *GAPDH*). **(b) ***BCoR-L1 *expression in LCLs from breast cancer families (normalised to *DIDO-1*). **(c) **Mean and standard deviation of *BCoR-L1 *expression in samples, grouped according to type of family cancer or *BCoR-L1 *genotype (normalised to *GAPDH*). **(d) **Mean and standard deviation of *BCoR-L1 *expression in samples, grouped according to type of family cancer or *BCoR-L1 *genotype (normalised to *DIDO-1*). *Subject also carries a *BRCA2 *mutation. ^#^Subjects from the same *BCoR-L1 *haplotype sharing family. *BCoR-L1*, BCL6 corepressor-like 1; *DIDO-1*, death inducer-obliterator 1; *GAPDH*, glyceraldehyde-3-phosphate dehydrogenase.

The intron 5 c.3608-156C>T variant was found only in members of the family who carried the exon 4 synonymous variant p.N172N (Figure 2). Due to its deeply intronic location, c.3608-156C>T is not predicted to have any functional effect. SpliceSiteFinder did not predict any changes to splicing as a result of this nucleotide substitution [37]. Furthermore, mRNA expression analysis (see below) of *BCoR-L1 *in two of the variant-carrying breast cancer cases and one wild-type non-breast cancer subject from this family revealed no evidence for consistent differences in expression in LCLs. Relative to the unaffected wild-type family control, one variant carrier displayed increased expression and the other displayed decreased expression (Figure 3a,b; subjects marked with #).

The two other variants detected in and around the *BCoR-L1 *coding region in this study, c.625G>A (p.G209S) and c.5075+21C>T, were found in similar frequencies in the control sample. Similarly, there were no major differences between groups when the study sample was divided into male breast, prostate, or *BCoR-L1 *haplotype sharing families. Although the exon 4 p.G209S variant is a missense amino acid substitution, this change is predicted by SIFT (Sorting Intolerant From Tolerant) to be 'tolerated' [38]. p.G209S is also located in a region of BCoR-L1 that is not thought to be involved in BRCA1 interaction or transcription repression [10]. Additionally, qRT-PCR analysis of *BCoR-L1 *expression in breast cancer cases carrying the p.G209S and c.5075+21C>T variants showed no differences when compared with controls (Figure 3c,d), with overlapping standard deviations for expression. Furthermore, both variants were present in individuals found to carry pathogenic *BRCA2 *mutations during the course of the study.

Overall, we detected very little variation in the coding region (and surrounding intronic region) of the *BCoR-L1 *gene in our population of familial cancer cases. The low level of variation detected in the *BCoR-L1 *gene is consistent with reports that the X chromosome carries very little variation when compared with autosomal chromosomes [39-41].

### *BCoR-L1 *expression analysis

To further investigate the role of *BCoR-L1 *in familial breast cancer and male cancers, we undertook qRT-PCR expression analysis on LCLs from subjects previously screened for *BCoR-L1 *coding region variation. Alteration of *BCoR-L1 *expression in LCLs from breast cancer-affected family members may indicate the presence of a regulatory mutation in a noncoding region of the gene. In addition, since various HDACs are aberrantly expressed in a number of cancers susceptible to treatment using HDAC inhibitors [42], including breast cancer [43], we speculated that BCoR-L1, given its association with HDACs, might also be abnormally expressed in breast cancer cases. We thus assessed *BCoR-L1 *expression in a range of breast, ovarian, and prostate cancer lines.

### *DIDO1 *expression in lymphoblastoid cell lines and cell lines

**Figure 4:**
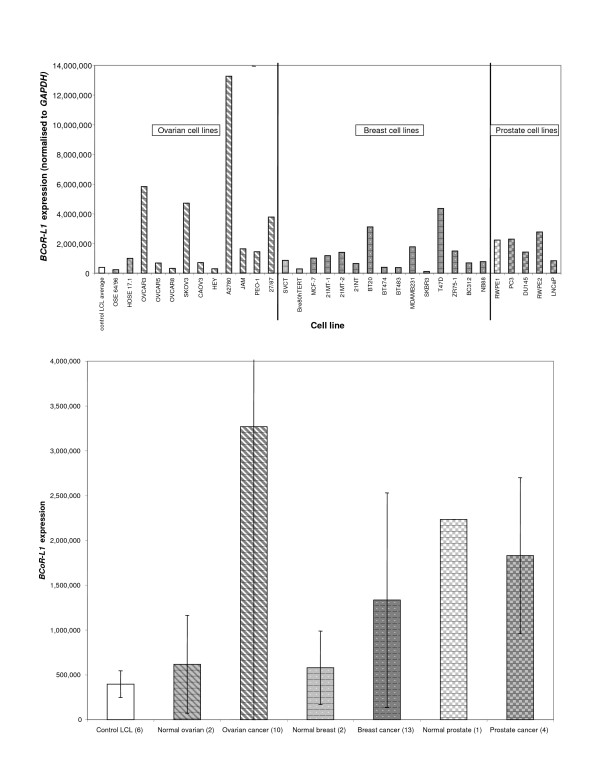
**Variation in control gene expression**. **(a) **Variation in control gene expression in lymphoblastoid cell lines. **(b) **Variation in control gene expression in cell lines. *DIDO-1*, death inducer-obliterator 1; *GAPDH*, glyceraldehyde-3-phosphate dehydrogenase; *UBC*, ubiquitin C.

To investigate the suitability of *DIDO1 *as an LCL expression control, we analysed expression of *DIDO1*, *GAPDH*, and *UBC *in all LCLs and cell lines tested (Figure 4a,b). Interestingly, we found that *DIDO1 *expression varies considerably less in LCLs than *GAPDH *or *UBC*, with *GAPDH *and *UBC *showing similar levels of expression variation (Figure 4a). The interquartile range for *DIDO1 *was 2.5-fold and 2.7-fold less than those for *UBC *and *GAPDH*, respectively. In cancer cell lines, all three expression control genes showed large ranges of expression levels across lines, with *GAPDH *perhaps showing the least variation (Figure 4b). Therefore, *DIDO1 *is an improved expression control for LCLs but has variability similar to the commonly used *GAPDH *control in normal and cancer cell lines.

### *BCoR-L1 *expression in lymphoblastoid cell lines from breast cancer families

Twenty-nine LCLs from breast cancer cases and their family members (23 families in total, including 14 with male breast cancer, 7 with prostate cancer, and 2 *BCoR-L1 *haplotype sharing families) were analysed for changes in *BCoR-L1 *expression when compared with LCLs from 6 healthy controls (Figure 3a–d). Although expression of *BCoR-L1 *appeared to be greatly variable, there were no apparent differences in expression levels between breast cancer cases and controls, nor were there any differences between groups when segregated by family cancer type (that is, male breast, prostate, and so on). Likewise, there was no indication of any association between *BCoR-L1 *genotype and expression. Skewed X chromosome inactivation data were available for a limited number of samples (*n *= 8). However, skewing did not correlate with *BCoR-L1 *expression or genotype (data not shown). Results correlating expression with sample source or sample genotype were similar using *GAPDH *(Figure 3a,c) or *DIDO1 *(Figure 3b,d) normalisation, although the decreased variability observed in normal controls for *DIDO1 *normalisation supported our earlier observations that *DIDO1 *is an improved control for LCL expression analysis. The standard deviation of expression in control LCLs was approximately 20% less for *DIDO1 *compared with *GAPDH*. Overall, it appears that *BCoR-L1 *expression is not altered in familial breast cancer cases, even for subgroups defined by male cancer type, and it is unlikely that there is any variation in the *BCoR-L1 *gene (detected or otherwise) which has a profound effect on expression.

### *BCoR-L1 *expression in cancer and normal cell lines

To assess a possible role for *BCoR-L1 *in tumourigenesis, we also analysed *BCoR-L1 *expression in various ovarian, breast, and prostate cancer and normal cell lines (Figure 1a,b). Most normal and cancer cell lines exhibited increased expression levels compared with LCLs. Once again, it was observed that *BCoR-L1 *expression is highly variable in both cancer and normal cell lines, with up to 13-fold differences in expression observed. There were no significant differences between the mean *BCoR-L1 *expression in normal cell lines compared with cancer cell lines, but individual ovarian and breast cancer cell lines showed significantly increased expression compared with the mean expression in normal cell lines. Markedly elevated levels of *BCoR-L1 *(*P *< 0.05) were observed for a total of 4/10 ovarian cancer cell lines (OVCAR3, SKOV3, A2780, 27/87; 4-fold to 13-fold upregulation compared with the HOSE17.1 normal ovarian epithelial control) and 2/13 breast cancer cell lines (BT20 and T47D; 3-fold and 4-fold upregulation compared with SVCT normal breast control). This was interesting, considering that skewed X inactivation has been reported in ovarian cancer cases [19]. It would also seem to suggest that dysregulation of expression in the form of *upregulation *may play a role in tumourigenesis. However, a study using a *BCoR-L1*-containing Affymetrix microarray (Affymetrix, Santa Clara, CA, USA) did not provide any evidence for *BCoR-L1 *deregulation in ovarian cancers [44]. Comparison of gene expression between 37 advanced-stage serous ovarian carcinomas and normal ovarian surface epithelium cytobrushings revealed that *BCoR-L1 *was not one of 1,191 genes that were significantly differentially regulated (defined as greater than or equal to 1.5-fold change in expression). This contrasts with the 4- to 13-fold increased expression we observed for specific cancer lines compared with normal ovarian epithelial cell lines.

There was no evidence that the hormone receptor status of each cell line correlated with these *BCoR-L1 *expression differences because the highly estrogen receptor (ER)- and progesterone receptor (PR)-positive breast cancer cell line T47D showed similar *BCoR-L1 *expression to BT20, a breast cancer cell line that does not express ER or PR [45]. In addition, the ovarian cancer cell lines OVCAR3 (ER-positive) and SKOV3 (ER-negative) expressed similar levels of *BCoR-L1 *[46]. Investigation of the X chromosome karyotype of these ovarian cancer cell lines did reveal a possible relationship with *BCoR-L1 *expression. Karyotype data were available for OVCAR3, SKOV3, OVCAR5, and OVCAR8 [47], with the *BCoR-L1*-overexpressing lines OVCAR3 and SKOV3 found to possess three copies of Xq and four X chromosomes, respectively. Conversely, OVCAR5 and OVCAR8 each possess only one X chromosome and express normal levels of *BCoR-L1*. Information on the X chromosome karyotype of breast cancer cell lines was limited, but there was no indication of an association between karyotype and *BCoR-L1 *expression [47,48]. The finding that *BCoR-L1 *expression tends to correlate with supernumary X chromosomes suggests that this overexpression probably does not contribute to carcinogenesis but may occur as a result of carcinogenesis. Although it is unknown whether the superfluous X chromosomes in OVCAR3 and SKOV3 are active, it has been reported that ovarian (and breast) tumours can possess multiple active X chromosomes [48-51].

## Conclusion

The aim of the present study was to attempt to elucidate a role for *BCoR-L1 *as a high-risk breast cancer predisposition gene by mutation screening of well-characterised non-*BRCA1/2 *familial breast cancer subjects who were selected to maximise the probability of identifying a mutation. We detected minimal variation in the coding region of *BCoR-L1*, which may imply the importance of maintaining the structural integrity of the BCoR-L1 protein. It is also unlikely that noncoding region variation in the *BCoR-L1 *gene is involved in breast cancer predisposition as we did not detect any significant changes in expression between cancer cases and cell lines and controls. The absence of pathogenic coding mutations or expression deregulation in this set of familial cases indicates that *BCoR-L1 *is extremely unlikely to be a major high-risk familial breast cancer predisposition gene. However, it is still possible that *BCoR-L1 *could be involved in breast cancer predisposition as a moderate- or low-penetrance risk gene, as has been found with *CHEK2 *[6], *BRIP1 *[7], and *PALB2 *[8,9] in large studies of *BRCAX *cases and controls. The involvement of *BCoR-L1 *in ovarian cancer may also be worthy of investigation, and further functional analysis of the BCoR-L1 protein will help to elucidate the involvement of BCoR-L1 in various essential pathways.

## Abbreviations

ATM = ataxia telangiectasia-mutated; BCoR-L1 = BCL6 corepressor-like 1; *BRCAX *= *BRCA1/2 *mutation-negative; DHPLC = denaturing high-performance liquid chromatography; *DIDO1 *= death inducer-obliterator 1; ER = estrogen receptor; ESE = exonic splice enhancer; *GAPDH *= glyceraldehyde-3-phosphate dehydrogenase; HDAC = histone deacetylase; kConFab = Kathleen Cuningham Foundation Consortium for Research into Familial Breast Cancer; LCL = lymphoblastoid cell line; LOH = loss of heterozygosity; PCR = polymerase chain reaction; PR = progesterone receptor; qRT-PCR = quantitative real-time polymerase chain reaction; *UBC *= ubiquitin C.

## Competing interests

The authors declare that they have no competing interests.

## Authors' contributions

FL carried out the screening of the gene and the LOH and expression analysis and drafted the manuscript. JA provided RNA from all of the breast and ovarian cell lines. GJM and GMP performed haplotype sharing analysis. CJB performed the X inactivation status analysis and drafted the relevant section of the manuscript. DBY contributed to the experimental study design and provided sequence data on *BCoR-L1*. kConFab recruited and collected subjects for the study. GC-T and KKK participated in the design and coordination of the study. ABS conceived the study, participated in its design and coordination, and helped to draft the manuscript. All authors read and approved the final manuscript.
